# Echafolta—Its Uses in Dental Surgery

**Published:** 1903-09-15

**Authors:** B. F. Thielen

**Affiliations:** Paris, Texas


### ECHAFOLTA—ITS USES IN DENTAL SURGERY.

B. F. THIELEN, D.D.S., PARIS, TEXAS.

Echafolta is derived from *echinacea augustafolia*, a western plant, native of Nebraska and other sections of the Northwest. In 1897, it was identified, and its reputed qualities brought before the profession by Lloyd Brothers and Professor John King, M.D. The credit for the discovery of the qualities of the drug belong, however, to Dr. H. F. C. Meyer, who had used it since 1870.

So convinced was Dr. Meyer of its efficacy in snakebite that, under professional care, he was willing to be bitten by a rattlesnake, feeling sure that the administration of echafolta would save his life. This drug is without doubt the most recent and valuable of the recent accessions to therapeutics. Few remedies not actively poisonous fulfill as wide a range of indications. Undoubtedly its basic action is on the fluids of the body—an antagonist of that state known as "blood depravation." Perhaps it can not be termed an antiseptic, an antizymotic, or an antiferment, yet it covers the field occupied by all the vegetable drugs of these classes.

The best term that we can apply to it is that of a corrective of blood dyscrasia. It antagonizes the influence, be it septic or otherwise, of low or morbid accumulations in the fluids or changes in the fluids themselves. Bad blood and its consequent adynamia are pre-eminently the conditions in which it is indicated. It exhibits marked anodyne properties, and in malignant carbuncle, septic ulcerations and abscesses, sloughing erysipelas, diphtheritic manifestations, stings and bites of insects and reptiles, its action is specific.

My attention was first called to this drug by an article in the *Dental Cosmos* of March, 1902. Dr. Hewitt, of Chicago, in speaking of the painless curetting of antrum, gave the formula of a topical application that he used, one constituent of which was echafolta. He described the drug and then reported a case where he had used it, which I will quote.

"A gentleman came to me six months ago, with a ranula under his tongue. A physician had opened it, and I believe his lancet was unclean or septic. At all events, he came to me, just sauntered through my doorway, saying, 'Doctor, I want you to treat me,' and stumbled onto the lounge. His tongue was swollen, his pulse was 130 to the minute, his temperature was over 100, and he looked as if he would not live three hours. I did not dare to send him over to the physicians, because I was afraid he would lose time and die. I gave him echafolta. I took two drachms and put it into four ounces of water. I applied the dose every fifteen minutes. I gave him two spoonfuls of this medicine every ten minutes. His face and neck down to the shoulders were swollen—a case of septicemia such as I have seldom met—but I persevered in this treatment, and next morning his pulse had gone down to about 80, and his temperature was about normal. The treatment was continued two days, and the man is living and says he owes his life to me."

A few days after reading this article I was talking to a friend, a physician, about a case that was giving him a great deal of trouble. His patient had received an injury to the middle finger of the right hand that necessitated amputation of that member, and although all necessary precautions as to asepsis were taken, the wound became infected, septicemia developed, and he was contemplating the amputation of the whole hand. I told him of what I had read concerning this drug, and he decided to try it.

He used it both locally and internally, and told me that his patient began to improve from the first dose, and made a good recovery without the loss of his hand.

A short while after this a gentleman came into my office, complaining of pain in his lower jaw, on the right side. An examination revealed the fact that the second and third molars on this side had been extracted. The gum was inflamed and there were several openings in the gum, through which pus would exude on pressure. His condition was diagnosed as necrosis, and an operation for the removal of the diseased bone was advised. After injecting a solution of eucain I made an incision through the gum to the bone, and with a curette removed all the necrosed tissue. Then the wound was thoroughly cleansed with warm water, after which

Echafolta........................................2 drachms,
Water, dist....................................3 ounces, was applied locally with a syringe three times a day for three days, and the patient required to take internally a teaspoonful of echafolta, 2 drachms dissolved in 4 ounces of distilled water every hour for three days. The wound healed nicely.

There was no sign of inflammation, and the recovery was immediate. Another case, that of a woman with an abscessed lower wisdom tooth, presented itself. The tooth was only partially erupted; her jaw was very much swollen, the adjacent tissues much inflamed, and there was a ragged incision in the gum, made by her family physician in an effort to let out the pus and relieve the pain.

I advised that she take an anesthetic and have the tooth removed, to which she consented, and the operation was performed. After she had come from under the influence of the chloroform, the wound was washed out as well as possible under the circumstances, an antiseptic wash prescribed, and she went home. The next morning she returned; had spent a wakeful night, and was still suffering from the pain. Upon examination the tissues were found much inflamed, her temperature was elevated, and a very foul odor emanated from the wound. I proceeded at once to wash it thoroughly, then applied echafolta, as in the above case, and prescribed it internally. She was told to return the following day, but did not, and in a few days had forgotten she ever had the trouble.

I use this drug wherever it is indicated, and have the first case to see where it has failed.—*Texas Journal.*

